# The Antimalarial Chloroquine Reduces the Burden of Persistent Atrial Fibrillation

**DOI:** 10.3389/fphar.2019.01392

**Published:** 2019-11-27

**Authors:** Catalina Tobón, Laura C. Palacio, Bojjibabu Chidipi, Diana P. Slough, Thanh Tran, Nhi Tran, Michelle Reiser, Yu-Shan Lin, Bengt Herweg, Dany Sayad, Javier Saiz, Sami Noujaim

**Affiliations:** ^1^MATBIOM, Universidad de Medellín, Medellín, Colombia; ^2^Molecular Pharmacology and Physiology Department, University of South Florida Morsani College of Medicine, Tampa, FL, United States; ^3^Department of Chemistry, Tufts University, Medford, MA, United States; ^4^Cardiology Department, University of South Florida Morsani College of Medicine, Tampa, FL, United States; ^5^Ci^2^ B, Universitat Politècnica de València, Valencia, Spain

**Keywords:** chloroquine, persistent atrial fibrillation, potassium inward rectifiers, I_KACh_, I_K1_

## Abstract

In clinical practice, reducing the burden of persistent atrial fibrillation by pharmacological means is challenging. We explored if blocking the background and the acetylcholine-activated inward rectifier potassium currents (I_K1_ and I_KACh_) could be antiarrhythmic in persistent atrial fibrillation. We thus tested the hypothesis that blocking I_K1_ and I_KACh_ with chloroquine decreases the burden of persistent atrial fibrillation. We used patch clamp to determine the IC_50_ of I_K1_ and I_KACh_ block by chloroquine and molecular modeling to simulate the interaction between chloroquine and Kir2.1 and Kir3.1, the molecular correlates of I_K1_ and I_KACh_. We then tested, as a proof of concept, if oral chloroquine administration to a patient with persistent atrial fibrillation can decrease the arrhythmia burden. We also simulated the effects of chloroquine in a 3D model of human atria with persistent atrial fibrillation. In patch clamp the IC_50_ of I_K1_ block by chloroquine was similar to that of I_KACh_. A 14-day regimen of oral chloroquine significantly decreased the burden of persistent atrial fibrillation in a patient. Mathematical simulations of persistent atrial fibrillation in a 3D model of human atria suggested that chloroquine prolonged the action potential duration, leading to failure of reentrant excitation, and the subsequent termination of the arrhythmia. The combined block of I_K1_ and I_KACh_ can be a targeted therapeutic strategy for persistent atrial fibrillation.

## Introduction

Atrial fibrillation (AF) is the most common heart rhythm abnormality and its incidence and prevalence are increasingly alarming. In addition to increasing mortality, AF is a known risk factor for stroke (Wolf et al., 1991), dementia (Liao et al., 2015; Singh-Manoux et al., 2017), and cardiac contractile dysfunction (Schotten et al., 2002). Pharmacological rhythm control therapy for AF remains attractive, however, when AF becomes persistent, pharmacotherapy is frequently ineffective (Zimetbaum, 2012).

Chloroquine, an antimalarial 4-aminoquinoline, has been suggested to have antiarrhythmic properties through unknown mechanisms (Burrell and Martinez, 1958; Harris et al., 1988), and was shown to have a safe cardiac electrophysiological profile (Wozniacka et al., 2006; Teixeira et al., 2014). Our previous work suggested that chloroquine terminates different forms of AF in animal models, in part, due to its ability to block potassium inward rectifiers (Noujaim et al., 2010; Filgueiras-Rama et al., 2012; Takemoto et al., 2018).

In persistent AF, the acetylcholine-activated inward rectifier potassium current I_KACh_ (molecular correlates are Kir3.1 and Kir3.4) was shown to be constitutively active and the inward rectifier potassium current I_K1_ (molecular correlates Kir2.1 and Kir2.3) has been shown to be upregulated (Voigt et al., 2010; Dobrev and Nattel, 2011). The constitutively active I_KACh_ could be considered as a background inward rectifier, which along with the increased I_K1_ may contribute to the shortening of the action potential duration and to create a substrate for the formation of stable, high frequency electrical rotors that maintain fibrillation (Atienza and Jalife, 2007; Noujaim et al., 2007). Consequently, blocking inward rectifiers could potentially be antiarrhythmic in the setting of persistent AF.

Recently, we used structural biology approaches to map the binding pocket of chloroquine in the intracellular domain of Kir3.1 (Takemoto et al., 2018). Chloroquine blocked I_KACh_ by binding at a specific site in the Kir3.1 intracellular ion permeation pathway (Takemoto et al., 2018). Previous work by us and others showed that chloroquine also blocks I_k1_ (Rodriguez-Menchaca et al., 2008; Noujaim et al., 2010). In this study, we tested in a patient with persistent AF whether chloroquine can reduce the arrhythmia burden, and we further explored the antiarrhythmic effects of chloroquine in a 3D model of the human atria with persistent AF.

## Methods

### Patch Clamp

Experiments were performed as described earlier (Noujaim et al., 2011; Takemoto et al., 2018). HEK293 cells stably co-transfected with Kir3.1 and Kir3.4 were generously provided by the Bayliss Laboratory (University of Virginia), and HEK293 cells stably transfected with Kir2.1 were generously provided by the Jalife Laboratory (University of Michigan). Currents were recorded using EPC 800 amplifier (HEKA Elektronik, Lambrecht/Pfalz), A/D converter (Digidata 1550B plus Hum Silencer, Molecular Devices, San Jose, CA), and the pClamp 10.6 PC software (Molecular Devices). Analysis was performed with Clampfit 10.6 (pClamp, Molecular Devices) and OriginPro software packages (version 2018, Microcal). The patch pipettes had a resistance of 2.5–3MΩ. After GΩ seal formation, whole cell recordings were performed at room temperature (around 24°C). For I_KACh_, the bath solution contained (in mM) 90 NaCl, 50 KCl, 1 CaCl_2_, 2 MgCl_2_, 10 HEPES, 10 glucose, and the pH adjusted to 7.4. The pipette internal solution contained (in mM) 100 K-aspartate, 10 NaCl, 40 KCl, 5 Mg-ATP, 2 EGTA, 0.1 GTP-Tris, and 10 HEPES at pH 7.2. For I_K1_, the bath solution contained (in mM) 148 NaCl, 0.4 NaH_2_PO_4_, 1 MgCl_2_, 5.4 KCl, 1.8 CaCl_2_, 5.5 glucose, 15 HEPES, pH 7.4, and the pipette internal solution contained (in mM), 150 KCl, 1 MgCl_2_, 5 EGTA, 5 HEPES, 5 phosphocreatine, 4.4 K_2_ATP, and pH 7.2. I_KACh_ and I_K1_ were measured as the 1mM BaCl_2_ sensitive current. Concentration–response curves and IC_50_ for currents inhibition at −140mV were determined with Prism 7 software using the standard variable slope equation *Y* = 1/(1 + 10^(^ *^LogIC^* ^50^^−^ *^X^* ^)^*^Slope^).

### Molecular Docking of Chloroquine Into Kir2.1 and Kir3.1

As we previously described, the intracellular domain of tetrameric Kir2.1 (PDB: 1U4F) (Pegan et al., 2005) and Kir3.1 (PDB: 1U4E) (Pegan et al., 2005) were used for the docking simulations. The SMILES string for chloroquine was obtained from Drugbank (Wishart et al., 2008) (Accession Number: DB00608). The Kir2.1 and Kir3.1 proteins, and chloroquine were prepared using the Protein Preparation Wizard and LigPrep in Schrodinger’s Maestro (Sastry et al., 2013), respectively. The protonation state of the drug was generated at a target pH of 7.0 in Epik (Shelley et al., 2007; Greenwood et al., 2010). The ligand was energy minimized with the OPLS_2005 force field (Shivakumar et al., 2010). A set of grids was created to map the proteins topologies before docking. The grids were generated using 60 × 60 × 60 points in the *x*, *y*, and *z* directions and centered around the aqueous channel with a spacing of 0.375 Å. Docking was performed with the Lamarckian genetic algorithm, with the population size set to 150, the number of generations set to 27,000 and the number of energy evaluations set to 2,500,000. Using Autodock 4.2 (Morris et al., 2009), one thousand runs were performed. The lowest energy poses from molecular docking were analyzed. Hydrogen bond analysis was performed using Visual Molecular Dynamics (Humphrey et al., 1996).

### Estimation of Blocking Ability of Chloroquine

A voxel grid was generated using the atomic coordinates of the lowest energy pose from molecular docking of the tetrameric Kir2.1 or Kir3.1 bound to chloroquine. In this grid, each voxel was a cube of 0.2 × 0.2 × 0.2 Å. The van der Waals radius of each atom (Iijima et al., 1987) was used to fill the corresponding number of cubes. The use of the discrete grid allowed for a probe to search the space in the aqueous ion-permeation pathway. The probes were Voxel-approximated spheres with radii ranging from 1.4 Å (the ionic radius of a bare K^+^ ion) to 3.9 Å in 0.1 Å increments. Three ångströms is the radius of a solvated K^+^ ion up to its first hydration shell (Mahler and Persson, 2012). Originating from the extracellular opening of the channel, the probes were pushed longitudinally through the channel towards the intracellular side. If, at any point in the pathway, the probe was unable to proceed further, the probe’s lateral position was adjusted in order to search all possible paths through the channel. The channel was considered blocked at a given probe radius if the probe was unable to find a path to move past chloroquine.

### Human Study

A 67 year old female with history of tobacco use, persistent AF (AF sustained for more than seven days, and less than a year), and no other significant past medical history including no known coronary artery disease, was enrolled in this study. Institutional Review Board approval was obtained at the University of South Florida, Tampa, USA, and the study conformed to the principles outlined in the Declaration of Helsinki. Prior workup revealed normal blood pressure. Her laboratory values showed normal kidney and liver function tests. Echocardiography was unremarkable for structural heart disease, with an ejection fraction >50%. The patient has been on Apixaban and Metoprolol, and continued to be on these medications during the study. The patient gave informed consent, was equipped with a Cardionet monitor, and received the chloroquine regimen for amebiasis treatment, consisting of 600 mg chloroquine base once daily for 2 days, followed by 300 mg daily for 12 days. She was continuously monitored for the duration of chloroquine treatment. A previous Holter monitor recording (ZIO XT Patch) for 12 days obtained two months prior, was reviewed and showed that the patient had been continuously in AF, with a burden of 100%. Additionally, 12 lead ECG was recorded 3 days before and on the day of chloroquine regimen initiation, confirming that the patient was in AF in both instances.

### Mathematical Simulations of Persistent Atrial Fibrillation in a 3D Model of Human Atria

The Courtemanche-Ramirez-Nattel membrane formalism (Courtemanche et al., 1998) was implemented to simulate the human atrial cell action potential. Cholinergic activity, a factor that promotes AF, was included in the model by implementing the following I_KACh_ equation developed by Kneller (Kneller et al., 2002):

$$I_{KACh}=\left(\frac{10}{1+9.13652/ACh^{0.477811}}\right)\left(0.0517+\frac{0.4516}{1+e^{V_{m}+59.53}/17.18}\right)(V-E_{K})$$

where *V* *_m_* is the membrane potential, *ACh* is the acetylcholine concentration and *E* *_K_* is the potassium equilibrium potential.

Based on experimental data (Workman et al., 2001; Van Wagoner, 2003), the cell model was modified in order to reproduce electrophysiological conditions of persistent AF: the maximum conductance of I_K1_ was increased by 100%, the maximum conductance of transient potassium current (I_to_) and delayed rectifier potassium current (I_Kur_) were decreased by 50%, and the maximum conductance of L-type calcium current (I_CaL_) was decreased by 70% and for the constitutively active I_KACh_, 5 nM of acetylcholine was simulated.

A realistic three-dimensional (3D) model of human atria including the main anatomical structures: left and right atria, twenty pectinate muscles, fossa ovalis, Bachmann’s bundle, crista terminalis, left and right appendages, pulmonary veins, caval veins, atrioventricular rings and coronary sinus, was previously developed (Tobon et al., 2013). The model also includes three different pathways for the inter-atrial conduction, specific fiber orientations in 42 different atrial regions, heterogeneous tissue conductivity, anisotropy ratios and heterogeneous cellular properties. The wall of the atrial model is a monolayer surface, except the Bachmann’s bundle and the pectinate muscles, which are solid structures. The model is composed of 52,906 hexahedral elements with a spatial resolution ranging from 300 to 700 µm.

The mathematical atrial cell model coupled with the 3D model was used to simulate a persistent AF episode. AF was generated with an S1–S2 stimulation protocol. S1 consisted of a train of ten stimuli with a basic cycle length of 1,000 ms, applied to the sinus node area, simulating sinus rhythm. After the last S1 stimulus, 6 ectopic S2 beats at 130 ms cycle length were delivered to the right superior pulmonary vein. Action potential propagation in the tissue was modeled using the monodomain reaction–diffusion equation:

$$\frac{1}{S_{v}}\nabla \cdot (D\nabla V_{m})=C_{m}\frac{\partial V_{m}}{\partial t}+I_{ion}-I_{stim}$$

where, *S* *_v_* corresponds to the surface-to-volume ratio (range from 0.0086 to 0.02), *D* is the conductivity tensor, *C* *_m_* is the membrane capacitance (100 pF), *I* *_ion_* is the total ionic current that crosses the cell membrane and *I* *_stim_* is the stimulus current (28 pA/pF). Equations were numerically solved using EMOS software (Heidenreich et al., 2010), which is a parallel code that implements the finite element method and operator splitting for solving the monodomain model. The time step was fixed to 0.001 ms. Simulation of 2 s of atrial activity required 32 h on a computing node with 12 dual core AMD Opteron Processors 2,218 clocked at 2.6 GHz.

Pseudo-unipolar atrial electrograms (EGMs) in the 3D model at 0.2 mm from the surface were simulated. The extracellular potential (*Φ* *_e_*) in the endocardial atrial surface was computed using the large volume conductor approximation

$$\phi_{e}(r)=-K\int \int \int \nabla^{\prime }V_{m}(r^{\prime })\cdot \nabla^{\prime }\left[\frac{1}{r^{\prime }-r}\right]dv$$

where *K* (−0.0398) is a constant that includes the ratio of intracellular and extracellular conductivities, ∇ʹ*V* *_m_* is the spatial gradient of transmembrane potential, *r* is the distance from the source point (*x, y, z*) to the measuring point (*x’, y’, z’*) and *dv* is the differential volume. EGMs at different points were visually inspected in order to analyze their morphologies as single, double or fractionated potentials. Double potentials were defined as EGMs with two negative or positive deflections and fractionated electrograms were defined as those exhibiting multiple (more than two) deflections. EGMs were processed with a 40–250 Hz band-pass filter, rectified and low-pass filtered at 20 Hz. Subsequently, spectral analysis of the signals was performed with a fast Fourier transform. The dominant frequency (DF) defined as the frequency corresponding to the highest peak of the power spectrum was calculated.

### Modeling the Effect of Chloroquine on Potassium Currents

**Figure 1:**
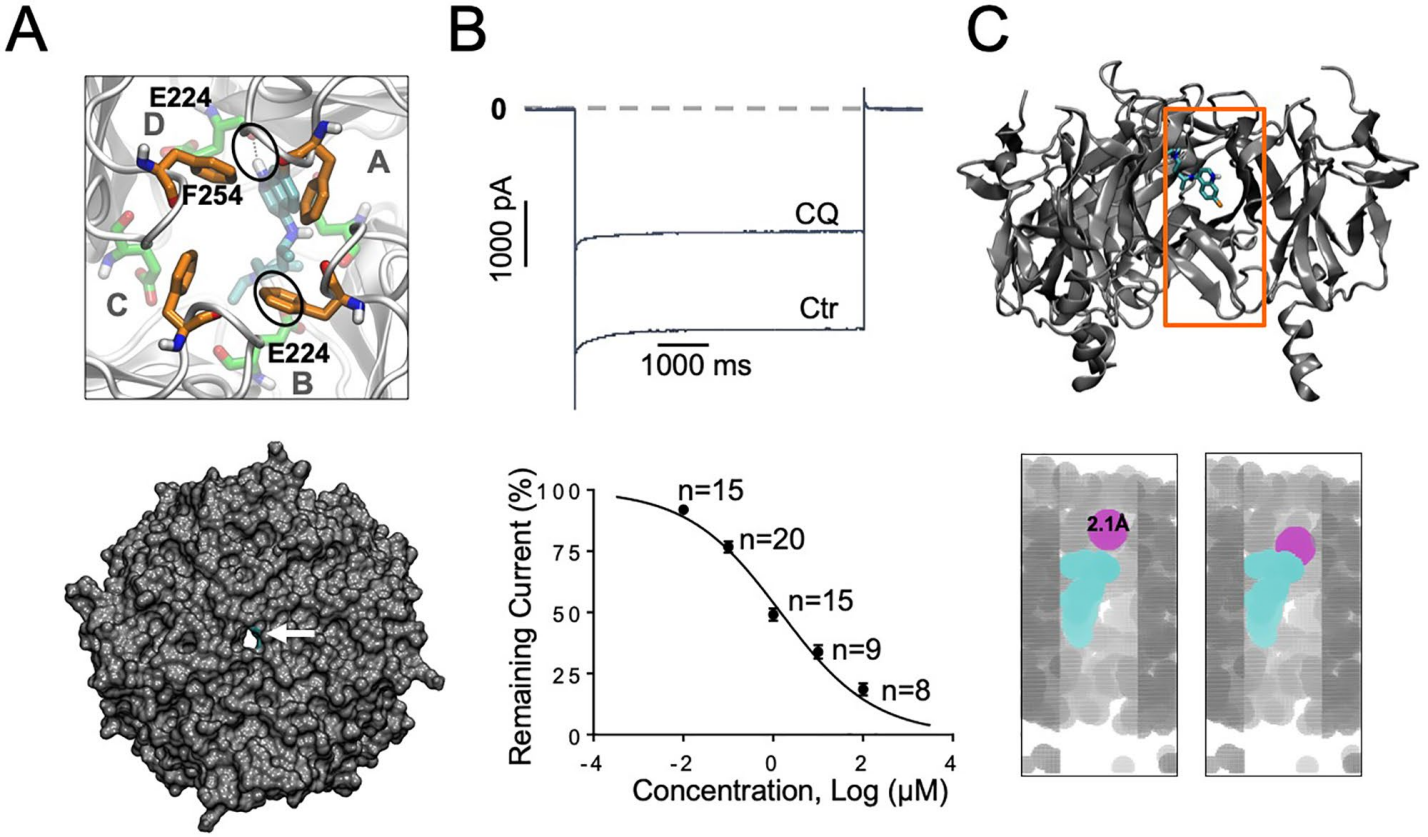
I_K1_ block with chloroquine. **(A)** Docking of chloroquine in the intracellular domain of Kir2.1. The lowest energy pose is shown. Chloroquine makes hydrogen bonds with residue E224 from subunit B and with E224 from subunit D (black circles). Bottom panel: Extracellular bird’s eye views of the van der Waals representation of the channel (gray) in complex with chloroquine (cyan). Chloroquine blocks the ion permeation pathway. **(B)** Barium sensitive I_K1_ traces in response to −140mV step pulses from a holding potential of 0 mV in the presence of 1 μM chloroquine (CQ). Concentration–response curves. IC_50_ for chloroquine block of I_K1_: 1.3 μM, Hill Slope = −0.42, R^2^=0.9. **(C)** Top panel: Longitudinal view of chloroquine bound to Kir2.1. Orange box denotes the voxelated part of the channel shown below. Bottom panel: Voxelation of Kir2.1 ion permeation pathway (grey) in complex with chloroquine (cyan). A probe (magenta) of radius 2.1 Å or larger, is blocked by chloroquine.

**Figure 2:**
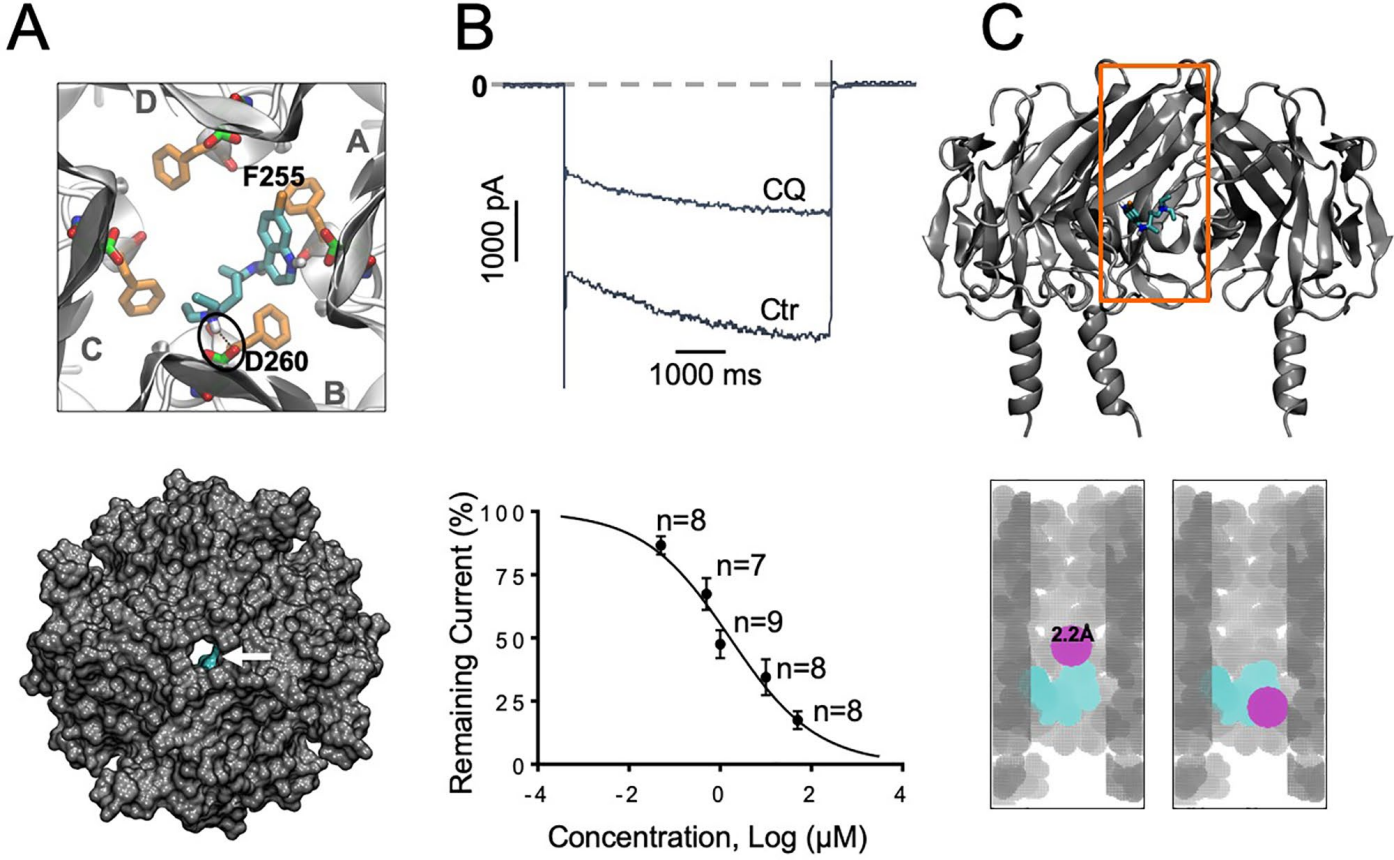
I_KACh_ block with chloroquine. **(A)** Docking of chloroquine in the intracellular domain of Kir3.1. The lowest energy pose is shown. The D260 and F255 residues from each of the four Kir3.1 subunits are shown in green and orange sticks respectively. The tertiary amine nitrogen of chloroquine (cyan sticks) forms a hydrogen bond (black circle) with the side chain of D260 in the B subunit, while the aminoquinoline ring of chloroquine is in close proximity to the phenylalanine ring of F255 in the A subunit. Van der Waals representations of the channel bound to the drugs (cyan) viewed from the extracellular side is shown in the bottom panel. **(B)** Barium sensitive I_KACh_ traces in response to −140mV step pulses from a holding potential of −20 mV in the presence of 1 μM chloroquine (CQ). **(B)** Concentration–response curves. IC50 for chloroquine block of I_KACh_: 1.2 μM, Hill Slope = −0.48, R^2^=0.74. **(C)** Top panel: Longitudinal view of chloroquine bound to Kir3.1. Orange box denotes the voxelated part of the channel shown below. Bottom panel: Voxelation of Kir3.1 ion permeation pathway (grey) in complex with chloroquine (cyan). A probe (magenta) of radius 2.2 Å or larger, is blocked by chloroquine.

To develop a basic model of the effect of chloroquine on the major potassium currents that this drug has been shown to block (I_KACh_, I_K1_, and I_Kr_) (Sanchez-Chapula et al., 2001), we used the steady state fraction of block (*f*), as done previously (Duarte M et al., 2013):

$$f=1/(1+IC_{50}/[C])$$

where *C* is the chloroquine concentration. The Hill equation was used to fit the Concentration–response relationships for chloroquine block. In this model, the channels kinetics were considered unchanged in the presence of the chloroquine. The IC_50_ for I_KACh_ and I_K1_ block by chloroquine were used from **Figures 1** and **2** and our earlier work (Noujaim et al., 2010) (0.97 µM for I_K1_ and 1.0 µM for I_KACh_). For I_Kr_, we used an IC_50_ of 2.5 µM as reported by Traebert et al in HEK293 cells (Traebert et al., 2004). The simulated fraction of block curve for I_KACh_ current fit well the experimental data shown in **Figures 1** and **2**, where (1 − *f*) · 100% is the remaining current. After 5 s of baseline AF simulation, chloroquine was applied at increasing concentrations, from 1.0 µM to 8.7 µM, in order to simulate ∼50% to ∼90% block of I_K1_ and I_KACh_, and from 1.0 µM to 25 µM, in order to simulate 30% to 90% block of I_Kr_. We investigated the effects of chloroquine on AF dynamics by blocking either I_K1_, I_KACh_ or I_Kr_ alone, or by blocking both I_K1_ and I_KACh_, or by blocking the three currents. Action potential duration at 90% repolarization (APD_90_) and the resting membrane potential (RMP) in a left atrial single cell were measured. All simulations were halted 2 s after chloroquine application.

## Results

### Chloroquine Block of I_K1_ and I_KACh_

Our earlier study using modeling, and protein NMR, suggested that chloroquine binds the intracellular domain of Kir3.1, at the level of amino acids F255 and D260 (Takemoto et al., 2018). Here, we conducted similar molecular docking simulations in order to compare the binding of chloroquine to Kir2.1 and Kir3.1. For each complex, one thousand docking runs were performed. The lowest energy poses for ligand–protein interactions were further studied. **Figures 1A** and **2A**, left panels, are zoomed in views of the tetrameric Kir2.1 (**Figure 1A**) and Kir3.1 (**Figure 2A**) channels, represented by gray ribbons, with the docked chloroquine depicted in cyan. In Kir2.1 (**Figure 1A**), the tertiary amine of chloroquine hydrogen-bonded to residue E224 of subunit B (black circles), and the aminoquinoline ring formed a hydrogen bond with the carbonyl oxygen of the diametrically opposed E224 of subunit D. The binding energy of chloroquine to Kir2.1 was –6.09 Kcal/mol. In Kir3.1 (**Figure 2A**), the tertiary amine of chloroquine hydrogen-bonded to residue D260 of subunit B (black circles), and the aminoquinoline ring was in proximity to the F255 ring of the adjacent subunit A. The binding energy of the ligand to the channel was −6.37 Kcal/mol. The Van der Waals representations of the Kir2.1 and Kir3.1 in complex with chloroquine are shown from the extracellular bird’s eye views (**Figures 1A** and **2A**, bottom panels). The ion permeation pathways are in the middle, and the white arrows point to chloroquine which is shown in cyan. We then used patch clamp to compare the IC_50_ for chloroquine block of I_K1_ (**Figure 1B**) and I_KACh_ (**Figure 2B**). Top panels are representative current traces in response to voltage steps in the presence of 1 µM chloroquine. In the concentration–response curves, the IC_50_s of I_K1_ and I_KACh_ block by chloroquine at −140 mV were 1.3 µM and 1.2 µM respectively. Subsequently, we voxelated the models for Kir2.1 and Kir3.1 in order to verify that in the docked structures, chloroquine is able to block the flow of potassium ions through the intracellular ion permeation pathway (**Figures 1C** and **2C**). The intracellular domains of Kir2.1 and 3.1 are shown in the longitudinal view, with the front subunit removed, and bound chloroquine (cyan sticks) is exposed. In Kir2.1, chloroquine binds closer to the G loop area, while in Kir3.1, chloroquine binds towards the intracellular mouth of the permeation pathway. The orange box denotes the voxelated part of the channels presented in the bottom panels of **Figures 1C** and **2C**. It was found that in Kir2.1, a probe (magenta sphere) of radius ≥2.1 Å was blocked by the drug, while probes with radii <2.1 Å passed through. In Kir3.1, a probe of radius ≥2.2 Å was blocked by the drug, while probes with radii <2.2 Å passed through. 3.0 Å is the radius of a solvated K^+^ ion up to its first hydration shell. These results are in agreement with the patch clamp experiments which showed similar IC_50_ for chloroquine block of Kir2.1 and Kir3.1.

### Chloroquine Treatment in a Patient With Persistent AF

**Figure 3:**
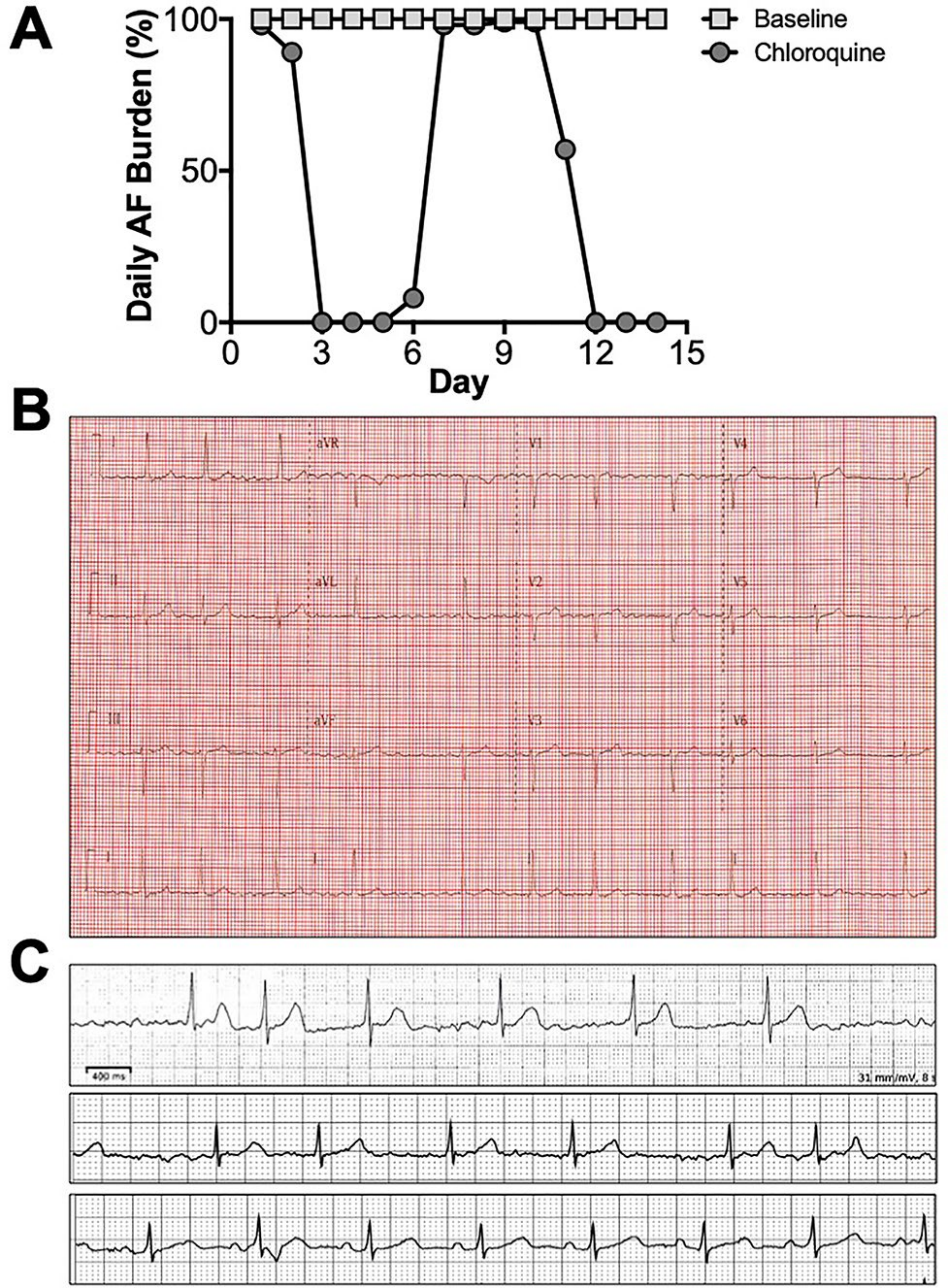
Chloroquine effects on persistent AF. **(A)** Daily AF burden for 14 days in Holter monitoring at baseline, and for 14 days upon initiation of oral chloroquine (amebiasis treatment regimen). **(B)** 12 lead ECG on the day of chloroquine initiation, confirming the presence of AF. **(C)** Holter ECG strips from baseline (top), right before treatment initiation (middle), and on the 3^rd^ day of treatment when AF converted to sinus rhythm (bottom).

As proof of concept, we tested in a patient with persistent atrial fibrillation, whether chloroquine can modify the AF burden. This 67 year old female was non-diabetic, non-hypertensive, a previous smoker, with no known structural heart or coronary artery disease but with known persistent AF. **Figure 3A** shows the daily AF burden extracted from Holter monitoring. At baseline, the patient was in uninterrupted, persistent AF. **Figure 3B** is a 12 lead ECG taken on the day of study initiation in order to confirm the presence of AF. After initiation of chloroquine treatment, AF burden was significantly reduced, with extensive periods of normal sinus rhythm, and the first conversion to normal sinus rhythm happened on the third day of treatment. **Figure 3C** shows Holter traces at baseline (top), immediately before treatment initiation (middle), where the patient was in AF, and during sinus rhythm on the third day of the oral chloroquine regimen (bottom). The patient did not have premature ventricular contractions while on chloroquine. The QT and QTc intervals while in AF, at the beginning of the study, were 418 ms and 428 ms respectively, and at the end of study, the QT and QTc intervals while in sinus rhythm were 450 ms and 461 ms. After the chloroquine regimen was completed, and upon follow up, the patient was back in atrial fibrillation as documented by a 12 lead ECG.

### Effects of Chloroquine on Persistent AF in a 3D Model of Human Atria

**Figure 4:**
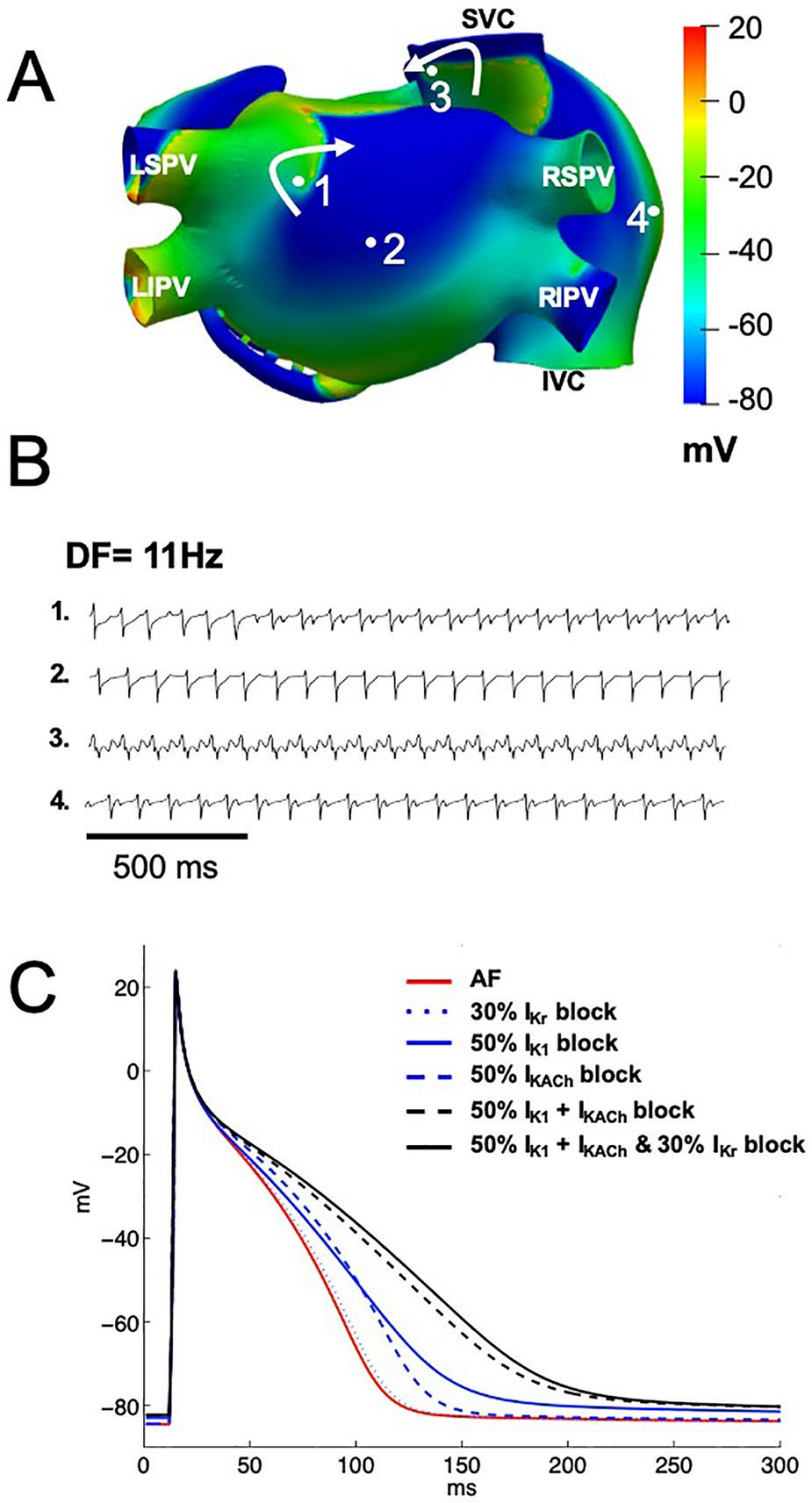
Computational simulations of persistent AF in a 3D model of the human atria. **(A)** Snapshots of membrane voltage during persistent AF maintained by 2 rotors (curved arrows indicate rotation direction) in the posterior left atrium and the superior vena cava (SVC). Numbered dots indicate the locations of EGM recordings. **(B)** EGM traces displaying single (trace 2), double (traces 1 and 4) and fractionated (trace 3) potentials with low amplitude and high frequency of activation (DF of 11 Hz). **(C)** Single cell left atrial action potentials, at baseline AF (red trace), when I_Kr_ (blue dotted line), I_K1_ (blue solid line), I_KACh_ (blue dashed line), I_K1_ and I_KACh_ together (black dashed line), or the three currents together (black solid line) were blocked by 1 μM of chloroquine. LIPV, and LSPV: left inferior and superior pulmonary vein. RIPV, and RSPV: right inferior and superior pulmonary vein. SVC, and IVC: superior and inferior vena cava.

A model of persistent AF was initiated by ectopic activity in the right superior pulmonary vein, and the arrhythmia was maintained by two stable rotors located in the posterior wall of the left atrium, near the left superior pulmonary vein and in the superior vena cava (**Figure 4A**). The calculated EGMs at sites 1, and 3, where the rotors were localized, showed double and fractionated potentials with low amplitude (**Figure 4B**). This occurred when the tip of the rotors pivoted at these locations, where the excitable but unexcited core resulted in multiple low amplitude deflections in the EGM. EGMs with single and double potentials were observed at sites 2 and 4 and were generated by wavefronts emanating from rotors and by wavebreaks at anatomical structures such as the crista terminalis and pectinate muscles. The DF was about 11 Hz (**Figure 4B**).

Chloroquine blocks I_K1_ and I_KACh_ with a similar IC_50_ of about 1 µM (0.97 µM for I_K1_ and 1.0 µM for I_KACh_), this is around the plasma concentration that is achieved in patients. Additionally, chloroquine blocks I_Kr_ with an IC_50_ of 2.5 µM. We thus tested if chloroquine block of either I_K1_, I_KACh_, I_Kr_ alone, or I_K1_ and I_KACh_ together, or the three currents together terminates persistent AF. **Figure 4C** shows single cell left atrial action potentials when chloroquine was applied at 1 µM in order to simulate ∼50% block of the I_K1_ and I_KACh_ currents and 30% block of I_Kr_. When I_Kr_ was blocked by 30% (blue dotted line), APD_90_ prolonged from 98 ms (baseline AF, red line) to 101 ms, without changes in the RMP (−84.6 mV). When either I_K1_ or I_KACh_ were blocked by ∼50% (blue solid and blue dashed lines), APD_90_ prolonged to 133 ms and 118 ms, with RMP values of −83.0 mV and −84.4 mV, respectively. When both I_K1_ and I_KACh_ currents were blocked by ∼50% (black dashed line), APD_90_ prolonged to 170 ms and the RMP slightly depolarized to −82.3 mV. When I_K1_, I_KACh_ and I_Kr_ currents were blocked by 1 µM of chloroquine (black solid line), the RMP slightly depolarized to −82.3 mV, but the APD_90_ prolonged to 179 ms.

When we increased the chloroquine concentration to 2.5 µM in order to simulate ∼70% block of the I_K1_ and I_KACh_ currents and 50% block of the I_Kr_ current, APD_90_ prolonged to 164 ms, 128 ms and 103 ms, with RMP of −81.2 mV, −84.3 mV and −84.6 mV, when I_K1_, or I_KACh_ or I_Kr_ was blocked respectively. When both I_K1_ and I_KACh_ were blocked by ∼70%, APD_90_ prolonged to 259 ms and the RMP depolarized to −78.6 mV. Finally, when the three currents were blocked by 2.5 µM of chloroquine, APD_90_ prolonged to 291 ms, which corresponds to a 197% increase.

**Figure 5:**
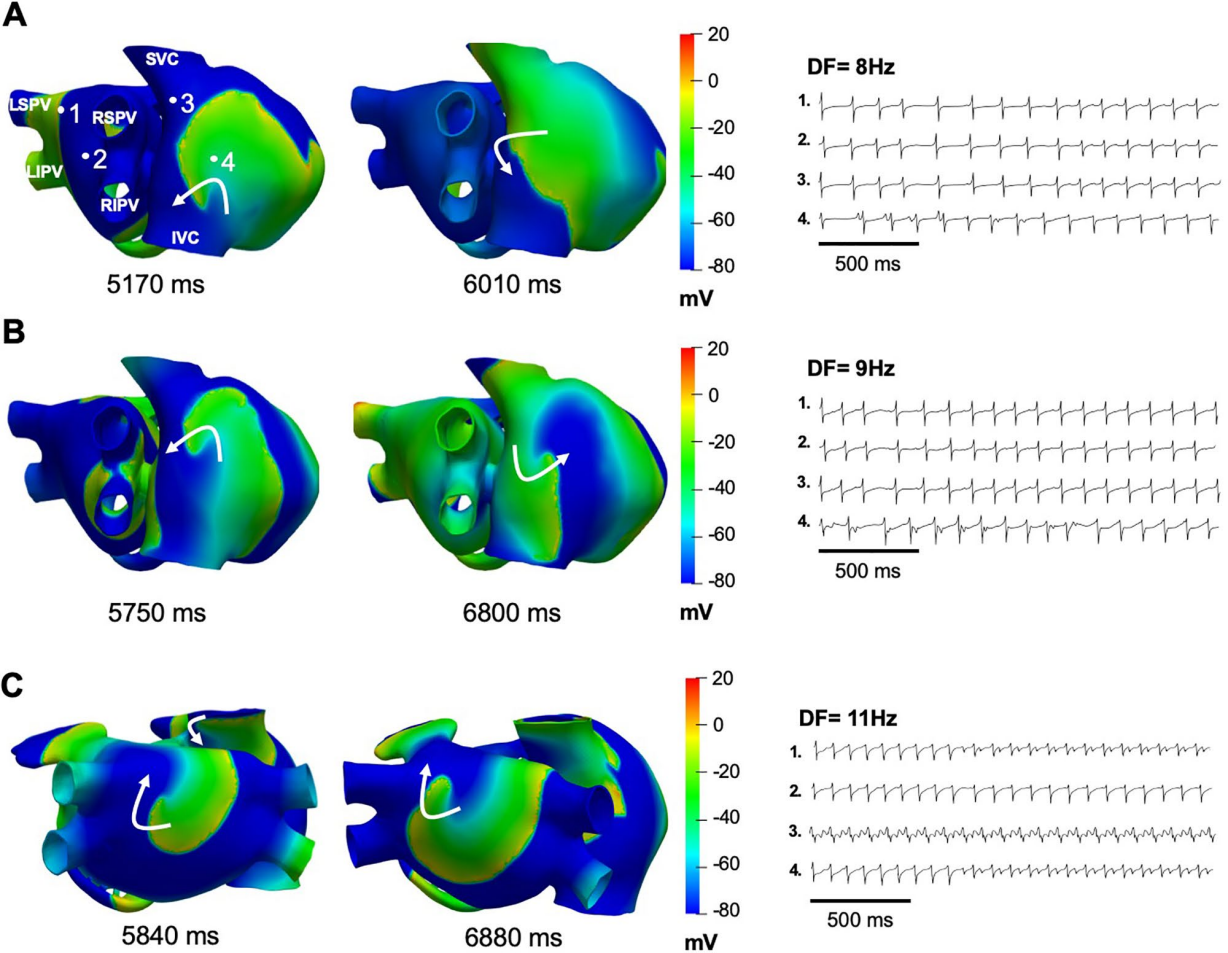
Simulations of I_K1_, I_KACh_, or I_Kr_ block on persistent AF. **(A)** Snapshot of the single re-entry maintaining atrial tachycardia when I_KACh_ was blocked by 70%, with the EGMs showing a DF of 8 Hz. **(B)** Snaphots of the single re-entry maintaining atrial tachycardia when I_K1_ was blocked by 90%, with the EGMs showing a DF of 9 Hz. **(C)** Snaphots of the 2 rotors maintaining AF when I_Kr_ was blocked by 90%, with the EGMs showing a DF of 11 Hz.

The two rotors maintaining AF at baseline continued to be present with up to 80% I_K1_ or 60% I_KACh_ block. In such scenarios, the rotor located in the posterior wall of the left atrium near the left pulmonary vein drifted to the inferior wall and the rotor located in the superior vena cava migrated to the free wall of the right atrium. Up to 90% block of I_Kr_ did not change the dynamics of the two rotors maintaining AF at baseline. However, when the block of I_K1_ reached 90% (**Figure 5A**) or I_KACh_ was blocked from 70 to 90% (**Figure 5B**), persistent AF converted into a stable reentrant tachycardia located at the free wall of the right atrium. In both cases, the EGMs presented single potentials generated by the wavefront emanating from the reentry and only in the posterior wall of the left atrium where the rotor wavefront pivots, some double EGMs generated by the passage of the rotor’s core were observed in this area (**Figures 5A, B**). The DF was reduced to values between 8 Hz and 9 Hz throughout the atria. **Figure 5C** shows that AF dynamics did not change appreciably when I_Kr_ was blocked by 90%.

**Figure 6:**
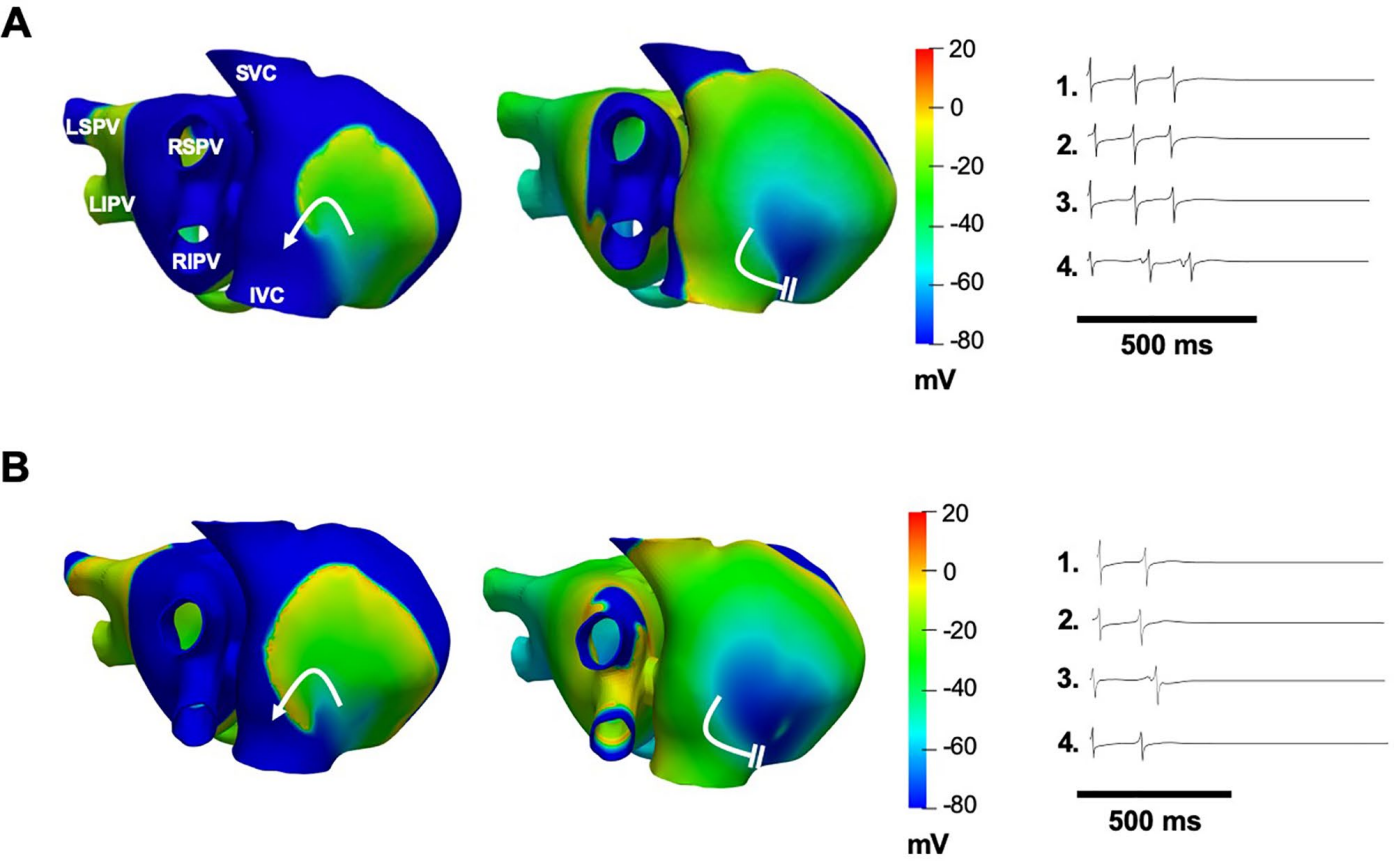
Simulations of 1 μM chloroquine terminating AF by dual block of I_K1_ and I_KACh_ by 50% **(A)**, or by block of I_Kr_ by 30% and block of I_K1_ and I_KACh_ by 50% **(B)**. Voltage maps are snapshots of the last rotation of the re-entrant wave, where AF terminates after the wavefront interacts with the wavetail (white parallel lines). Curved white arrows: rotation direction. EGMs show arrhythmia termination.

When the effects of chloroquine on both I_K1_ and I_KACh_ together were simulated, termination of persistent AF was achieved with ∼50% block of I_K1_ and I_KACh_ (**Figure 6A**). AF terminated 600 ms after the drug application, due to wavefront–wavetail interaction. Similarly, when the effects of chloroquine on the three currents together were simulated, termination of persistent AF was achieved with ∼50% block of I_K1_ and I_KACh_ and 30% block of I_Kr_ (**Figure 6B**). AF terminated 380 ms after the drug application. AF terminated earlier when higher concentrations of chloroquine were simulated.

## Discussion

Our results showed that the aminoquinoline chloroquine blocks I_K1_ and I_KACh_V by binding a pocket in the Kir2.1 and Kir3.1 channels intracellular ion permeation pathways. Moreover, chloroquine significantly reduced the burden of persistent AF in an otherwise healthy female patient. This was likely due to the ability of the drug to prolong the action potential duration, leading to a slowing down of the arrhythmia’s dominant frequency, and subsequent failure of reentrant excitation due to wavefront–wavetail interaction.

Our molecular modeling suggested that chloroquine blocks I_K1_ and I_KACh_ by binding to a pocket in the intracellular water filled vestibule of Kir2.1 and Kir3.1. The drug appeared to hydrogen bond with electronegative residue D260 in Kir3.1, and E224 in Kir2.1, in proximity to residue F255 in Kir3.1 and the equivalent Kir2.1 residue F254.

At baseline AF, our simulations showed EGMs with double and fractionated potentials with low amplitude and high frequency when the tip of the rotors pivoted on the recording points (pivot point). Experimental and clinical studies of AF have documented intracardiac polymorphic EGMs in high frequency areas with fibrillatory conduction (Ryu et al., 2006; Kalifa et al., 2006; Narayan and Krummen, 2012; Narayan et al., 2012a; Narayan et al., 2012b; Narayan et al., 2012c; Narayan et al., 2012d) and fractionated EGMs when the rotor core passes at the recording point (Zlochiver et al., 2008). When I_K1_ or I_KACh_ were individually blocked with increasing concentrations of chloroquine AF organized into reentrant atrial tachycardia due to APD prolongation. EGMs with single potentials in the atria were observed, characteristic of EGMs recorded during atrial tachycardias (Ryu et al., 2006). Double potentials were generated by the passage of the reentry core at the recording point in the free wall of the right atrium. It has been reported that atrial potentials consisting of two or more deflections can be recorded during atrial tachycardias in sites of earliest activation such as the crista terminalis (De Groot and Schalij, 2006). When only I_Kr_ was blocked, AF dynamics did not change (**Figure 5C**) because I_Kr_ block had a minimal effect on the APD (**Figure 4C**). While it has been reported that chloroquine is an I_Kr_ blocker (Sanchez-Chapula et al., 2002; Traebert et al., 2004), and can prolong the QT interval (White, 2007; Stas et al., 2008), in our simulations I_Kr_ block did not contribute substantially to the atrial antiarrhythmic properties. This could be in part due to the lower expression of hERG channels in the atria compared to the ventricles (Pond et al., 2000). When both I_K1_ and I_KACh_ (**Figure 6A**) or I_K1_, I_KACh_ and I_Kr_ (**Figure 6B**) were blocked by 1 µM chloroquine, AF terminated.

The oral chloroquine regimen which we administered to a patient with persistent AF consisted of 600 mg chloroquine base once daily, for 2 days, followed by 300 mg once daily for 12 days. Therapeutic doses of chloroquine typically result in plasma concentrations around 1 µM (Karunajeewa et al., 2010), within the range of IC_50_ of I_KACh_ and I_K1_ block by the drug. The cardiac safety of chloroquine when used correctly is well documented. For instance, from cardiac electrophysiological safety standpoint, chloroquine is safe. This was shown in a large cohort of patients with systemic lupus erythematosus who have been treated with chloroquine for an average of 8 years. Chloroquine was found to be not only electrophysiologically safe, but to also have antiarrhythmic activity in those patients with autoimmune disease (Wozniacka et al., 2006; Teixeira et al., 2014). Chloroquine poisoning occurs with ingestion of more than 5 g of chloroquine per dose (Riou et al., 1988).

Presently, the treatment of persistent AF remains inadequate (Rietbrock et al., 2008; Shukla and Curtis, 2014). Clinical studies demonstrated that antiarrhythmics, or ablation strategies do not result in complete freedom from AF, thus antiarrhythmic drug therapy remains an important line of defense. With currently used antiarrhythmics, the rate of conversion to sinus rhythms is not optimal (Zimetbaum, 2012). Additionally, toxicities including life threatening arrhythmogenesis are a significant risk in antiarrhythmic rhythm control pharmacotherapy. Nevertheless, pharmacological maintenance of sinus rhythm offers secondary end point benefits such as improvement in left ventricular function, walking distance, in addition to atrial size reduction (Chung et al., 2005; Hagens et al., 2005; Rienstra et al., 2005). Hence, there is a need to improve the antiarrhythmic armamentarium, and to generate novel antiarrhythmics that are safe and that can reduce the burden of persistent AF or restore sinus rhythm. Based on this study and those of others, chloroquine appears to have antiarrhythmic properties in patients with AF (Burrell and Martinez, 1958; Harris et al., 1988). On the other hand, chloroquine’s known side effects include QT prolongation (White, 2007; Stas et al., 2008), and retinal and gastrointestinal adverse reactions (Al-Bari, 2015). Therefore, using rational design to discover novel inward rectifier blockers based on small aminoquinolines such as chloroquine, but with an improved safety profile, could offer new avenues in the search of new pharmacotherapies for AF.

The combination of 3D virtual models of atrial fibrillation with modeling of ion channel block by small molecules opens the door for the simulation of antiarrhythmic drug effects, leading the way for the development of a more effective and safer generation of anti AF agents.

## Limitations

Atrial remodeling in persistent AF is complex, with many changes occurring from the transcriptional to post-translational levels (Anisimov and Boheler, 2003; Volkova et al., 2005). In addition, autonomic, anatomical, sarcolemmal, and subsarcolemmal electrophysiological remodeling has been described in persistent AF, resulting in slow and discontinuous electrical propagation, and aberrant excitation–contraction coupling, as well as increased perpetuation of AF (Kumar et al., 2012). For instance, increased fibrosis (Koura et al., 2002), reduced I_CaL_, increased background inward rectifiers currents (I_K1_ and constitutively active I_KACh_) in addition to mitochondrial (Moslehi et al., 2012), and calcium handling abnormalities (Vest et al., 2005) are present in persistent AF. Such changes can play a significant role in creating the complex structural and functional substrates which promote the initiation and maintenance of high speed rotors responsible for atrial fibrillation. Therefore, it not unlikely that chloroquine’s effect is not exclusively due to the block of I_KACh_ and I_K1_. At the therapeutic chloroquine plasma concentration, which is around 1 µM, the drug blocks about 14% of the I_Na_ current, 7% of the I_CaL_, and 9% of I_Ks_ (Sanchez-Chapula et al., 2001). It thus becomes conceivable that the multi-target effect of chloroquine contributed to its antiarrhythmic properties in persistent AF.

Although the electrophysiological remodeling conditions that we used in our simulations can reproduce the action potential phenotype observed in patients with persistent AF, as mentioned above, they do not take into account the significant structural remodeling that is found in the chronically fibrillating atria, which increases the complexity of the arrhythmia. Additionally, our numerical results were obtained using a specific virtual atria model that lacks fibrosis, albeit it includes a great number of anatomical and morphological details including electrophysiology, anatomy, fiber direction, anisotropy, and heterogeneity. Although functional reentries and rotors have been widely reported as maintenance mechanisms of AF, future works must include structural remodeling in order to address how chloroquine may be antiarrhythmic when ionic and structural remodeling are the driving mechanisms of persistent AF.

The simulated results may be atrial cell model-dependent. For instance, other human atrial models show a more triangular action potential and have a flatter restitution curve, however, all models introduce ionic remodeling in a similar way, resulting in effects on the action potential that are similar to what our simulations suggest. These models generally include decreases in I_to_, I_CaL_ and I_Kur_, and increased I_K1_. More recently, AF models that also account for the remodeling of intracellular Ca^2+^ handling have been developed (Grandi et al., 2011; Skibsbye et al., 2016; Colman et al., 2017; Bai et al., 2018). However, the simpler I_to_/ I_CaL_/ I_Kur_/ I_K1_ approach of persistent AF modeling remains commonly used, and we do not think that the use of a more complex model would substantially change the main results of our work. For modeling the effects of I_Kr_ block by chloroquine, we did not perform a concentration–response curve of the current block by the drug. We relied instead on the literature reported IC_50_ of 2.5 µM (Traebert et al., 2004), and thus, it is possible that variations around this value could affect the simulation results.

Two studies have reported that a benzopyrene derivative (Podd et al., 2016) and a benzamide related compound (Walfridsson et al., 2015) selectively inhibited I_KACh_. However, the drugs failed in patients with paroxysmal AF and atrial flutter (Walfridsson et al., 2015; Podd et al., 2016). This could be because I_KACh_ is not constitutively active in paroxysmal AF (Voigt et al., 2007) and mechanistically, atrial flutter is very different from AF (Olshansky, 2004). Consequently, inhibiting I_KACh_ as part of an antiarrhythmic pharmaco-strategy should be reserved for the patient population in which the current is known to play a role. On the other hand, it is possible that chloroquine would reduce the burden of paroxysmal AF. It was shown that in patients with paroxysmal AF, a left/ right atrial gradient in I_K1_ exists (Voigt et al., 2010). This gradient could contribute to the ionic mechanism of paroxysmal AF, and therefore block of I_K1_ by chloroquine might be antiarrhythmic. Chloroquine might have limited antiarrhythmic effects in atrial flutter since its mechanisms are distinct from those of AF (Olshansky, 2004).

This is a proof of concept study which showed that chloroquine reduced persistent AF burden in a single patient. I_K1_ and I_KACh_ block is proposed as a plausible mechanism for this reduction as suggested by the numerical simulations. Further studies are needed in order to demonstrate a relation between I_KACh_ and I_K1_ block and reduction of persistent AF burden.

## Data Availability Statement

All datasets generated for this study are included in the article/supplementary material.

## Ethics Statement

The studies involving human participants were reviewed and approved by USF IRB. The patients/participants provided their written informed consent to participate in this study.

## Author Contributions

Designed Research: CT, LP, BC, DPS, TT, NT, MR, Y-SL, BH, DS, JS, and SN. Analyzed data: CT, LP, BC, DPS, Y-SL, BH, DS, JS, and SN. Wrote manuscript: CT, LP, BC, DPS, BH, JS, and SN.

## Funding

This work was supported in part by National Institutes of Health grants R21HL138064, R01HL129136, by the Dirección General de Política Científica de la Generalitat Valenciana (PROMETEO 2016/088), and by the ACM SIGHPC/Intel Computational & Data Science fellowship.

## Conflict of Interest

The authors declare that the research was conducted in the absence of any commercial or financial relationships that could be construed as a potential conflict of interest.
